# Analysis of naphthoquinone derivatives as topoisomerase I inhibitors using fragment based QSAR

**DOI:** 10.1186/1758-2946-5-S1-P22

**Published:** 2013-03-22

**Authors:** Bastikar Virupaksha, Gupte Alpana, Khadke Prashant, Deshpande Uday, Desideri Alessandro

**Affiliations:** 1 Department of Bioinformatics, JJT University, Rajasthan, India; 2 Rajiv Gandhi Institute of Biotechnology and IT, Bharti Vidyapeeth, Pune, Maharashtra, India; 3 Department of Structural Biology, University of Rome Tor Vergata, Rome, Italy

## 

In this study an attempt was made to understand the structural requirements for Topoisomerase I (Topo I) inhibition using a novel Group based QSAR (GQSAR) or fragment based QSAR technique. Here we combined the GQSAR technology with conventional 2D and 3D QSAR to derive GQSAR models for various reported naphthoquinone derivatives. Various regression models such as Multiple Regression (MRA), Partial Least Square (PLS) and Principal Component Analysis (PCA) as well as k-Nearest neighbor (k-NN) QSAR were used to develop several combined 2D and 3D GQSAR models. The GQSAR analyses revealed the importance of Geometrical topological indices and Baumann's alignment independent topological descriptors along with dipole moment and other general descriptors like HBonddonor and XYHydrophilic etc for governing the activity variation. Further the GQSAR showed that chemical variation like presence of substituted double bonded C atom separated from oxygen by 6 bonds and HBonddonor count are highly influential for achieving highly potent Topo I inhibitors. The Naphthoquinone derivatives having 2-CH(OX)-(CH2CH=CMe2)-5,8-dihydroxy-1,4-naphthoquinone substitutions are most important fragments for the inhibitory activity. In addition the k-nearest neighbor classification model resulted in 3 important descriptors like moment of inertia, quadrapole and hydrogen count. The developed models are interpretable with good statistical and predictive significance and can be used for guiding ligand modification for development of potential new Topo I inhibitors. From the present study it can be seen that the substitutions made on 2-CH(OX)-(CH2CH=CMe2)-5,8-dihydroxy-1,4-naphthoquinone position can result in better Topo I inhibitors.

